# Prenatal Folic Acid Supplements and Offspring’s Autism Spectrum Disorder: A Meta-analysis and Meta-regression

**DOI:** 10.1007/s10803-021-04951-8

**Published:** 2021-03-20

**Authors:** Xian Liu, Mingyang Zou, Caihong Sun, Lijie Wu, Wen-Xiong Chen

**Affiliations:** 1 Division of Birth Cohort Study, Guangzhou Women and Children’s Medical Center, Guangzhou Medical University, Guangzhou, China; 2 Department of Children’s and Adolescent Health, College of Public Health, Harbin Medical University, Harbin, China; 3 Department of Neurology, Guangzhou Women and Children’s Medical Center, Guangzhou Medical University, Guangzhou, China

**Keywords:** Autism spectrum disorder, Folic acid, Prenatal, Meta-analysis, Meta-regression

## Abstract

We systematically reviewed the evidence on the association between maternal folic acid supplementation and the risk of offspring’s autism spectrum disorders (ASD). A total of 10 studies with 23 sub-studies (9795 ASD cases) were included. Folic acid supplementation during early pregnancy was associated with a lower risk of offspring’s ASD [OR 0.57, 95% CI 0.41–0.78]. The consumption of a daily amount of at least 400 μg folic acid from dietary sources and supplements, was associated with a reduced risk of offspring ASD [OR 0.55, 95% CI 0.36–0.83]. Critical effective maternal folic acid supplementation strategies, such as intake timing and intake dosage, may aid the reduction in the risk of offspring ASD. This meta-analysis provided new insights for the prevention of offspring’s ASD.

**Supplementary Information:**

The online version contains supplementary material available at 10.1007/s10803-021-04951-8.

## Introduction

Autism Spectrum Disorders (ASD) are characterized by representative impairments in social relatedness and verbal and non-verbal communication, combined with restrictive, repetitive patterns of behavior (APA, 2013). The prevalence of ASD had risen significantly from 13.4 per 1000 children in 2010 (CDC, 2014), 15.3 in 2012 (Christensen et al., 2018), 17.0 in 2014 (Baio et al., 2018) to 27.9 in 2016 (Xu et al., 2019) in the USA. A recent meta-analysis indicated that the prevalence of ASD in China was 26.5/10,000, which demonstrated an increasing trend (Liu et al., 2018) although it was significantly lower than the reported prevalence abroad. The impairments of ASD can severely impact learning and social functioning that may persist into adulthood (Cheuk et al., 2011). Given that ASD is persistent disabling neural developmental disorders from early childhood, it had posed significant burdens on society and the economy (Baxter et al., 2015; Leigh & Du, 2015).

The variation in ASD occurrence is mostly due to genetic factors (Bai et al., 2019). However, environmental risk factors have been the focus of considerable attention in recent years (Schmidt et al., 2019; Surén et al., 2013). The hypothesis that the etiology of ASD is jointly mediated by genetic predispositions and environmental risk factors (Chaste & Leboyer, 2012; Gao et al., 2020; Ijomone et al., 2020; Kim et al., 2019; Lyall et al., 2017; Saxena et al., 2020) has been widely accepted. Environmental factors may trigger predisposing hereditary high-risk gene modifications during fetal development (Oldenburg et al., 2020; Saxena et al., 2020). The pervasiveness of folate’s role in metabolism, nervous system function, and as a precursor of S-adenosyl-methionine (SAM), which is the folate donor for DNA methylation, suggests that there may be an association between maternal folate levels during pregnancy and ASD mediated by DNA methylation (Saxena et al., 2020).

Folate is an essential water-soluble substance of the vitamin B family, which is present in natural foods, synthetic forms such as folic acid supplements, and food fortification products (Crider et al., 2012; Mastroiacovo & Addis, 2006). As a crucial 1-carbon source, folate plays critical roles in cellular pathways such as DNA, RNA, and protein methylation DNA synthesis (Crider et al., 2012). Before pregnancy, folic acid supplementation is recommended for all women of the reproductive age in order to decrease the risk of neural tube defects (MRC Vitamin Study Research Group,^1991^; McGarel et al., 2015; Valera-Gran et al., 2014). Fortification of food products was also implemented in the United States and Canada (De Wals et al., 2007; Pfeiffer et al., 2019) but not in other countries, such as Sweden, Norway, Denmark, and Israel (Devilbiss et al., 2017; Levine et al., 2018; Surén et al., 2013). Folic acid supplements during preconception may reduce the risk of autism and severe language delay in children (Roth et al., 2011; Surén et al., 2013). Abnormalities in folate metabolism may play a role in the occurrence of ASD (Frye et al., 2017, 2018; Saha et al., 2019; Shaik Mohammad et al., 2016).

With the rising increase in prevalence and poor prognosis of ASD (Maenner et al., 2020), it is imperative to enhance the prevention and treatment of this disease. Maternal use of folic acid supplements serves as a potentially crucial modifiable factor for fetal neurodevelopment. If maternal folic acid supplements can decrease the incidence of ASD, they can be used clinically in the prevention and treatment of ASD. Therefore, the potential of maternal use of folic acid supplements during pregnancy to prevent offspring ASD has been of increasing interest. Evidence derived from several studies on the association between maternal folic acid supplements and the prevention of ASD is inconclusive (Levine et al., 2018; Li et al., 2018; Surén et al., 2013; Virk et al., 2016). A case-cohort study from Israel, including 45,300 children, demonstrated that maternal exposure to folic acid supplements was associated with a lower risk of offspring's ASD. The information on folic acid supplement exposure was extracted from the Prescription Register, which significantly reduced recall bias (Levine et al., 2018). Levine et al., also assessed the preventive effect of maternal exposure to folic acid supplements during pregnancy for offspring’s ASD with and without intellectual disability (ID) (Levine et al., 2018). In the prospective Norwegian cohort study, folic acid intake from 4 weeks before to 8 weeks after conception was associated with a reduced risk of offspring's ASD (Surén et al., 2013). However, other studies (Li et al., 2018; Virk et al., 2016) reported a null association between maternal folic acid supplementation and ASD risk. A limited number of meta-analysis studies reported controversial findings regarding the association between maternal folic acid supplements and offspring ASD (Guo et al., 2019; Wang et al., 2017). Several limitations are present in the previous systematic reviews as follows: (Guo et al., 2019; Wang et al., 2017): The included ASD cases partly diagnosed by screening scales (Jiang et al., 2016; Sun et al., 2016), the extracted data were from an inappropriate study (Al-Farsi et al., 2013) without exploring the association between maternal folic acid intake and offspring ASD in the Wang’s review (Wang et al., 2017); the inclusion of studies (Strøm et al., 2018; Virk et al., 2016) from duplicate sources, an unclear distinction regarding ORs, RRs and HRs (Levine et al., 2018) following processing of the data, and misclassifications regarding the FA supplementation form (Surén et al., 2013) in another review (Guo et al., 2019); furthermore, high heterogeneity was noted without further exploration of meta-regression and sensitivity analysis in both reviews (Guo et al., 2019; Wang et al., 2017). Therefore, a comprehensive, critical, and updated review of the evidence is urgently required. In addition, crucial questions regarding the sensitive timing windows, suitable dosage, and appropriate supplement forms for maternal folic acid intake remain unexplored.

Therefore, a comprehensive systematic review and meta-analysis was conducted with the rigorous inclusion criteria to explore the association between maternal folic acid supplements and the risk of offspring's ASD. We also conducted a series of sensitive analysis and meta-regression to determine whether supplement timing, dose condition, supplementary mode, and folic acid food fortification moderated effect sizes. Given the public health and economic burden of autism, understanding the sensitive window and dose of maternal folic acid intake may efficiently and precisely improve prevention for the development of ASD in the offspring.

## Methods

### Literature Search and Selection Strategy

According to the Preferred Reporting Items for Systematic reviews and Meta-analysis (PRISMA) (Moher et al., 2009) and Meta-analysis Of Observational Studies in Epidemiology (MOOSE) guidelines (Stroup et al., 2000), we performed a systematic literature search in PubMed, EMBASE and Cochrane Library from their inception till January 31, 2020. Search terms relating to autism (e.g. 'autism, autism spectrum', 'autistic', 'autism spectrum disorders', 'autism spectrum disorder', 'autistic spectrum disorders’ 'autistic spectrum disorder', 'Asperger', 'Asperger's', 'Asperger's syndrome', "Asperger’s disorder”,’autistic spectrum’, ‘pervasive developmental disorder’,’pervasive developmental disorders’, ‘disintegrative disorder’,’Rett syndrome’) and folic acid (e.g. ‘Folate’, ‘Folacin’, ‘Folvite’, ‘B9’, and ‘Pteroylglutamic Acid’) were combined according to the principles of Boolean logic (using AND, OR, or NOT). The results were limited to human studies and publications written in English. No restrictions were defined on publication date or research location.

### Study Selection

Two independent reviewers conducted screening and selection by reading the titles and abstracts of all studies. The full texts were reviewed as required. The senior reviewer was consulted when consensus on eligibility could not be achieved. The inclusion criteria included the following: (1) cohort study or case–control study; (2) report of the maternal folic acid supplements during the prenatal period and ASD diagnosis in the offspring diagnosed at the age of two or following the observational outcomes; (3) determination of ASD diagnosis based on the Diagnostic and Statistical Manual of Mental Disorders IV/5 Version (DSM-IV/5), International Classification of Diseases 8th /9th/10th Version (ICD-8/9/10) or structured interviews.

### Data Extraction and Quality Assessment

A Strengthening the Reporting of Observational Studies in Epidemiology (STROBE)-based pre-designed form (Hörnell et al., 2017) was used for data extraction. The information was extracted and included the following: author, year of publication, study design, study location, sample size, study population, measures of ASD, mode of folic acid intake, timing of folic acid intake, dosage of folic acid intake, outcome, age of children and risk estimates (e.g., risk ratios [RRs], odds ratios [ORs], hazard ratios [HRs]). In case the original study reported data on several exposures, the information on each contributing factor was retrieved separately. If the effect size for the association of interest was not reported in the study, the ORs were calculated from raw data. The senior reviewer was involved in the discussion when discrepancies were observed between the two previous reviewers.

Two independent reviewers undertook quality assessment according to the Newcastle–Ottawa Scale (NOS) (Stang, 2010; Wells et al., 2015). The senior reviewer resolved the discrepancies on quality assessment to reach a consensus. The NOS contains eight items, classified into the three following domains: selection, comparability, development (cohort studies), or exposure (case–control studies). The NOS score ranges from 0 up to 9 (Stang, 2010; Wells et al., 2015). A total score of ≥ 8 indicated a high quality, a 5–7 a moderate quality, and ≤ 4 a low quality.

### Statistical Analysis

Initially, the association between the maternal folic acid supplement and the risk of offspring’s ASD outcome was examined using random-effects models. The result obtained following pooling of every single study’s estimate was reported as ORs with 95% CIs. Secondly, subgroup analyses were conducted to assess differences in the association between the maternal folic acid supplement and the risk of offspring’s ASD by the aforementioned approach regarding the timing of folic acid intake, the dose of folic acid intake, and the supplementary modes of folic acid.

Statistical heterogeneity among studies was assessed using I^2^ statistics. I^2^ values of 25, 50, and 75% were regarded as low, moderate, and high heterogeneity, respectively (Higgins et al., 2003). A funnel plot and Egger test were conducted to present the publication bias. The Galbraith plot was performed for heterogeneity analyses. Based on the Galbraith plot's heterogeneity source, the sensitivity analyses were further conducted to exclude high heterogeneity studies. Influence analyses (so-called Leave-one-out sensitivity analyses) were performed by repeatedly removing one study to confirm that the meta-analysis results were not affected by any individual study. To test our results' robustness, we also repeated the primary analyses using the updated quality effects (QE) model (Doi et al., 2015), which is especially adept at handling heterogeneity. The adjusted ORs and RRs with 95% CIs were pooled separately by the QE model.

Meta-regression was performed to explore potential factors responsible for heterogeneity. Confounders including the study design, study location, timing of folic acid intake and supplementary modes of folic acid were selected mainly based on theoretical, practical, and empirical association with folic acid supplementation effects on the offspring’s ASD. Data analyses were conducted in Stata software (version 14.2; Stata Corp) and MetaXL software. A 2-sided P < 0.05 was considered to be statistically significant.

## Results

### Search Results

**Fig. 1:**
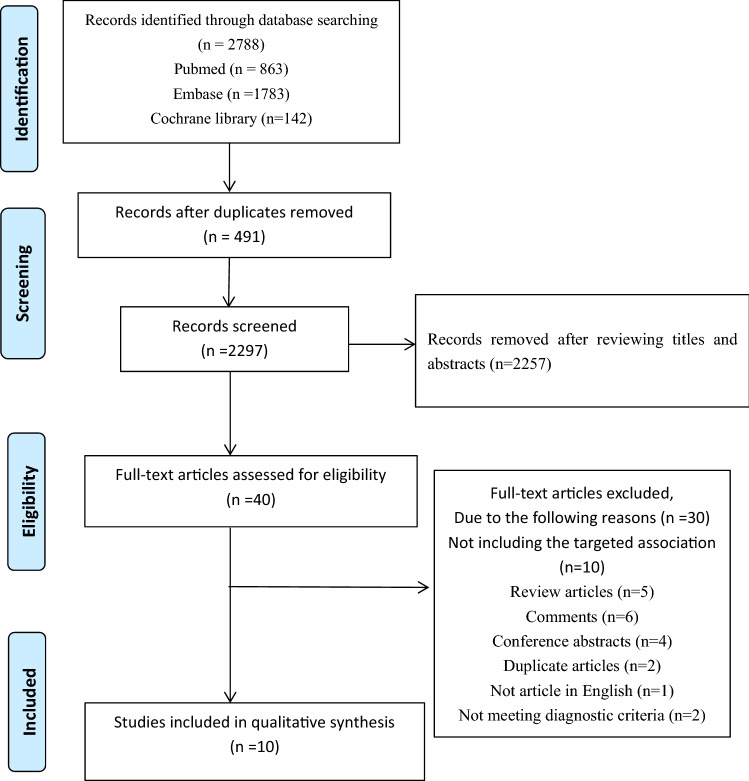
Flow diagram of studies selected for inclusion

A total of 2788 articles were identified from the selected database. There were 2297 unique records were present following removal duplicates. Of these, 2257 records were excluded following screening titles and abstracts. The full texts of 40 articles were reviewed. A total of 30 articles were excluded, since they did not meet the inclusion criteria based on the study design. Finally, ten articles (studies) were included in the review. A flow diagram of the study selection is shown in Fig. 1.

### Characteristics of Included Studies

The study characteristics of the ten included studies (Devilbiss et al., 2017; Levine et al., 2018; Li et al., 2018; Nilsen et al., 2013; Schmidt et al., 2012, 2017, 2019; Surén et al., 2013; Tan et al., 2020; Virk et al., 2016) with 23 sub-studies including 9795 ASD cases were summarized in Table 1. Of these, six were cohort studies (Devilbiss et al., 2017; Levine et al., 2018; Nilsen et al., 2013; Schmidt et al., 2019; Surén et al., 2013; Virk et al., 2016), and the other four were case–control studies (Li et al., 2018; Schmidt et al., 2012, 2017; Tan et al., 2020). Four out of ten studies were from European countries (Devilbiss et al., 2017; Nilsen et al., 2013; Surén et al., 2013; Virk et al., 2016), while the other six studies were derived from other countries such as the USA (the United States of America, n = 3) (Schmidt et al., 2012, 2017, 2019), Israel (n = 1) (Levine et al., 2018) and China (n = 2) (Li et al., 2018; Tan et al., 2020). The timings of folic acid intake were categorized into before pregnancy (folic acid exposure period occurred the period from 12 weeks before pregnancy to the start of pregnancy), early pregnancy (folic acid exposure period occurring at the period from the start of pregnancy to 12 weeks after the start of pregnancy), during pregnancy (no specific pregnancy and folic acid exposure period occurring at the period from 270 days before childbirth up to the date of delivery) and before pregnancy to early pregnancy (from 12 weeks before pregnancy to 12 weeks after the start of pregnancy). In terms of timing of folic acid intake, five sub-studies were included with folic acid intake before pregnancy to early pregnancy (Surén et al., 2013; Tan et al., 2020; Virk et al., 2016), whereas 11 sub-studies with folic acid intake were also included that examined early pregnancy (Devilbiss et al., 2017; Schmidt et al., 2012, 2017, 2019). A total of 3 sub-studies with folic acid intake focused on the period prior to pregnancy (Levine et al., 2018; Li et al., 2018). Moreover, three sub-studies with folic acid intake reported data during pregnancy (Levine et al., 2018; Li et al., 2018) and one sub-study with folic acid intake explored the period before and/or during pregnancy (Nilsen et al., 2013). The doses of folic acid intake were examined and ten sub-studies were included with a detailed consumption of an amount of folic acid intake (Schmidt et al., 2012, 2017, 2019; Tan et al., 2020). A total of 13 sub-studies were included without a detailed dose of folic acid intake (Devilbiss et al., 2017; Levine et al., 2018; Li et al., 2018; Nilsen et al., 2013; Schmidt et al., 2012; Surén et al., 2013; Virk et al., 2016). Based on modes of folic acid intake, folic acid supplements were classified as folic acid intake with other nutrients (n = 18 sub-studies) (Levine et al., 2018; Li et al., 2018; Schmidt et al., 2012, 2017, 2019; Surén et al., 2013; Virk et al., 2016) and as folic acid intake (n = 5 sub-studies) (Devilbiss et al., 2017; Levine et al., 2018; Nilsen et al., 2013; Tan et al., 2020).

**Table 1:** Characteristics of included studies in the systematic review and meta-analysis

Authors	Study design	Sample size (ASD case)	Study population	Measures of ASD	Mode of folic acid intake	Timing of FA intake	Outcomes	Dose of FA intake
Surén P et al.(2013)	Prospective cohort study	AD:85,176(114) AS:30,117(48) PDD-NOS:59,192(91)	The Norwegian Mother and Child Cohort Study, Norway	Diagnosed criteria: DSM-IV/ICD-10 ADI-R ADOS Age 3.3 to 10.2 years	Folic acid intake with other nutrients	Before and early pregnancy	AD,AS,PDD-NOS	No detail dose
DeVilbiss et al.(2017)	Prospective cohort study	94,684 (2123)	The Stockholm youth cohort, Sweden	Diagnosed criteria: DSM-V/ICD-10 Age: 4 to 15 years	Folic acid intake only	Early pregnancy	ASD	No detail dose
Virk et al.(2016)	Longitudinal population-based cohort study	19,042 (300)	Danish National Birth Cohort of pregnant women, Denmark	Diagnosed criteria: ICD-10 Age: 8.1–11.4 years	Folic acid intake with other nutrients	Before and early pregnancy	ASD	No detail dose
Schmidt et al.(2019)	Prospective cohort study	FA with 80–800 μg/day,149(50); FA with 805–4800 μg/day, 94(32); FA with ≥ 600 μg/day,181(55)	Markers of Autism Risk in Babies: Learning Early Signs, California, US	Diagnosed criteria: ADOS Age: a mean (SD) age of 36.5 (1.6) months	Folic acid intake with other nutrients	Early pregnancy	ASD	80–800 μg/day; 805–4800 μg/day; ≥ 600 μg/day
Levine SZ et al. (2018)	Case-cohort study	Before pregnancy, 45,300(572); During pregnancy, 45,300(572)	A case-cohort study established by linking health care registers from the Meuhedet health care, Israel	Diagnosed criteria: ICD-8R/ICD-9 Age:10–15 years	Folic acid intake with other nutrients; Folic acid intake alone	Before pregnancy and during pregnancy	ASD (ASD with ID, ASD without ID)	No detail dose
Schmidt et al.(2012)	Case–control study	no detail dose, 534(334); FA with ≤ 500 μg/day, 211(149); FA with 500 to < 800 μg/day, 90(61); FA with 800-1000 μg/day, 175(110); FA with > 1000 μg/day, 169(98)	The Childhood Autism Risks from Genetics and the Environment, California, US	Diagnosed criteria: SCQ ADOS ADI-R Age: 24 and 60 months	Folic acid intake with other nutrients	Early pregnancy	ASD	no detail dose; ≤ 500 μg/day; 500–800 μg/day; 800–1000 μg/day; > 1000 μg/day
Schmidt et al.(2017)	Case–control study	800 μg/day: 676(394) 600 μg/day: 676(394)	The Childhood Autism Risks from Genetics and the Environment, California, US	Diagnosed criteria: SCQ; ADOS; ADI-R Age:2–5 years	Folic acid intake with other nutrients	Early pregnancy	ASD	800 μg/day; 600 μg/day
Li et al.(2018)	Case–control study	Before pregnancy, 656(322); During pregnancy, 675(344)	Autism Clinical and Environmental Database, China	Diagnosed criteria: DSM-IV-TR Age:3–6 years	Folic acid intake with other nutrients	Before pregnancy and during pregnancy	ASD	No detail dose
Tan et al. (2019)	Case–control study	617 (416)	A total of 617 children were included in the study	Diagnosed criteria: DSM-5 Mean age:4.47 years	Folic acid intake only	Before and early pregnancy	ASD	400 μg/day
Nilsen et al. (2013)	A nationwide registry cohort study	507,856(2072)	The nationwide population, Norway (1999–2007)	Diagnosed criteria: ICD Mean age:7.0 years	Folic acid intake	before and/or during pregnancy	ASD	No detail dose

*ASD* autism spectrum disorder, *AD* autistic disorder, *AS* asperger syndrome, *PD-NOS* pervasive developmental disorder–not otherwise specified, *FA* folic acid, *CI* confidence interval, *DSM- IV/5* the diagnostic and statistical manual of mental disorders IV/5, *ICD-8R/9/10* international classification of diseases, eighth revision / ninth/tenth, *ADOS* autism diagnostic observation schedule-generic, *ADI-R* autism diagnostic interview-revised, *SCQ* social communication questionnaire, *ID* intellectual disability

### Quality of Included Studies

Table 2 demonstrates the quality assessment of the included studies. Ten studies were assessed for quality, according to the Newcastle–Ottawa scale. One of them was classified as high quality (Surén et al., 2013), while eight studies had moderate quality (Devilbiss et al., 2017; Levine et al., 2018; Nilsen et al., 2013; Schmidt et al., 2012, 2017, 2019; Tan et al., 2020; Virk et al., 2016), and the remaining one study exhibited low quality (Li et al., 2018).

**Table2:** Quality assessment of the included studies by the improved Newcastle–Ottawa scale

Study	Design	Selection	Comparability	Exposure/outcome	Total scores
Surén et al. (2013)	Cohort study	★★★★	★	★★★	8
DeVilbiss et al. (2017)	Cohort study	★★★★	★	★★	7
Virk et al. (2016)	Cohort study	★★★★	★	★★	7
Schmidt et al. (2019)	Cohort study	★★★★	★	★★	7
Schmidt et al. (2012)	Case–control	★★★★	★	★	6
Levine et al. (2018)	Cohort study	★★★★	★	★★	7
Schmidt et al. (2017)	Case–control	★★★★	★	★	6
Li et al. (2018)	Case–control	★★	★	★	4
Tan et al. (2019)	Case–control	★★	★	★★	5
Nilsen et al. (2013)	Cohort study	★★★★	★	★★	7

### Meta-analysis

**Fig. 2:**
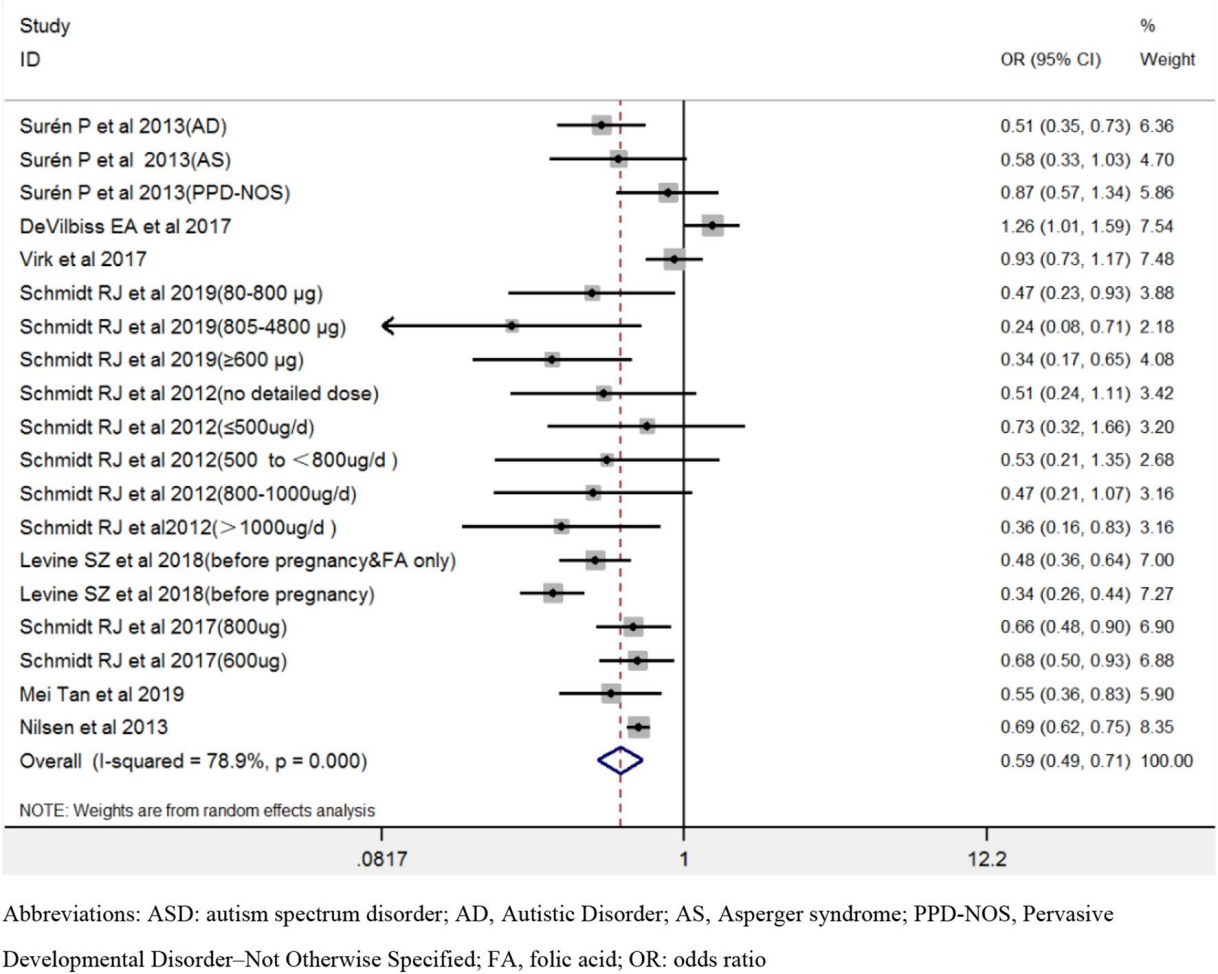
The forest plot of the association between maternal folic acid supplements exposure during the prenatal period and offspring’s ASD

Figure 2 showed the pooled estimates for maternal folic acid supplements' effect during the prenatal period on the offspring’s ASD using all available data. Maternal folic acid supplements during the prenatal period lowered the risk of offspring’s ASD [OR 0.57, 95% CI 0.46–0.72].

**Fig. 3:**
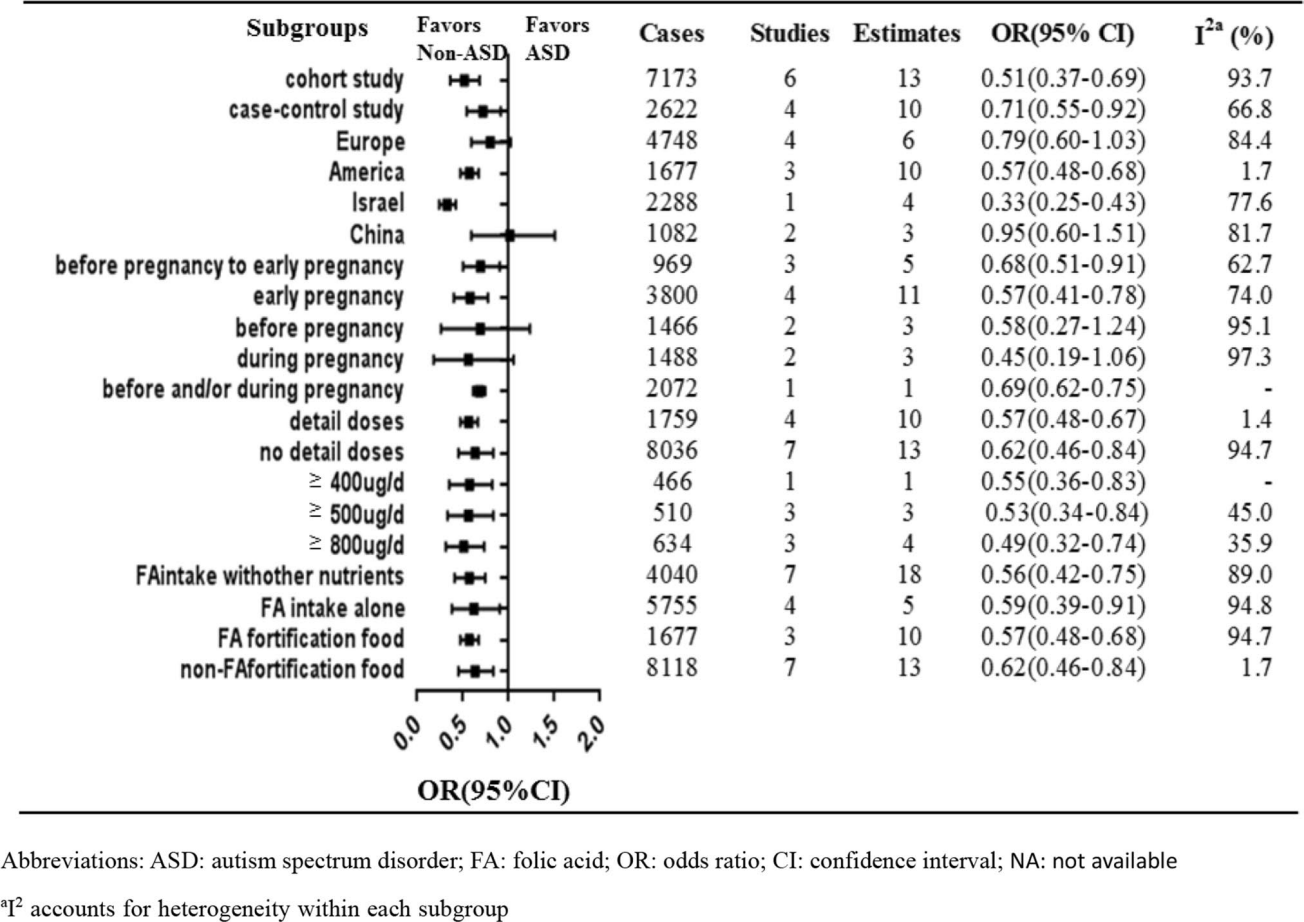
Subgroup analysis for maternal use of folic acid supplements and risk of offspring’s ASD

Figure 3 showed the subgroup analysis for maternal use of folic acid supplements and the risk of offspring′s ASD. Consistent results were reported both in prospective studies (Devilbiss et al., 2017; Levine et al., 2018; Nilsen et al., 2013; Schmidt et al., 2019; Surén et al., 2013; Virk et al., 2016) (thirteen estimates) [OR 0.51, 95% CI 0.37–0.69] and in case–control studies (Li et al., 2018; Schmidt et al., 2012, 2017; Tan et al., 2020) (ten estimates) [OR 0.71, 95% CI 0.55–0.92]. The results of subgroup analyses by location were basically consistent with the overall results as follows: in Europe (Devilbiss et al., 2017; Nilsen et al., 2013; Surén et al., 2013; Virk et al., 2016) (six estimates) [OR 0.79, 95% CI 0.60–1.03], in America (Schmidt et al., 2012, 2017, 2019) (ten estimates) [OR 0.57, 95% CI 0.48–0.68], and in Israel area (Levine et al., 2018) (four estimates) [OR 0.33, 95% CI 0.25–0.43]. The Studies from China (Li et al., 2018; Tan et al., 2020) (three estimates) presented an overall non-significant OR of 0.95 [95% CI 0.60–1.51]. In terms of the timing of folic acid intake, three studies (Surén et al., 2013; Tan et al., 2020; Virk et al., 2016) (five estimates) reported that folic acid supplementation was obtained from before pregnancy to early pregnancy resulted in an OR of 0.68 [95% CI 0.51–0.91], while four studies (Devilbiss et al., 2017; Schmidt et al., 2012, 2017, 2019) (eleven estimates) were included describing folic acid intake in early pregnancy with an OR of 0.57 [95% CI 0.41–0.78]. Two studies (Levine et al., 2018; Li et al., 2018) (three estimates) of folic acid supplementation before pregnancy had an OR of 0.58 [95% CI 0.27–1.24] and other two studies (Levine et al., 2018; Li et al., 2018) (three estimates) of folic acid supplementation during pregnancy had an OR of 0.45 [95% CI 0.19–1.06]. One study (Nilsen et al., 2013) (one estimate) that examined folic acid supplementation before and/or during pregnancy exhibited an OR of 0.69 [95% CI 0.62–0.75]. Four studies (Schmidt et al., 2012, 2017, 2019; Tan et al., 2020) (ten estimates) with detailed folic acid dose had a pooled OR of 0.57 (95% CI 0.48–0.67), while other seven studies (Devilbiss et al., 2017; Levine et al., 2018; Li et al., 2018; Nilsen et al., 2013; Schmidt et al., 2012; Surén et al., 2013; Virk et al., 2016) (thirteen estimates) without detailed folic acid dose resulted in a pooled OR of 0.62 [95% CI 0.46–0.84] (Fig. 3). Four studies with exact folic acid dose were included in the meta-analysis to explore further the dose–effect (Schmidt et al., 2012, 2017, 2019; Tan et al., 2020). The respective risk of the estimates between maternal folic acid intake and offspring’s ASD were 0.55 [95% CI 0.36–0.83], 0.53 [95% CI 0.34–0.84], and 0.49 [95% CI 0.32–0.74], when the consumption folic acid was higher than 400, 500, and 800 μg/day, respectively. For supplementary modes, four studies (Devilbiss et al., 2017; Levine et al., 2018; Tan et al., 2020; Nilsen et al., 2013) (four estimates) with folic acid intake resulted in a pooled OR of 0.59 [95% CI 0.39–0.91], and seven studies (Levine et al., 2018; Li et al., 2018; Schmidt et al., 2012, 2017, 2019; Surén et al., 2013; Virk et al., 2016) (eighteen estimates) with folic acid and other nutrients intake resulted in a pooled OR of 0.56 [95% CI 0.42–0.75] (Li et al., 2018). Maternal folic acid supplements reduced the risk of offspring’s ASD in countries with (three studies with ten estimates) (Schmidt et al., 2012, 2017, 2019) (OR 0.62, 95% CI 0.46–0.84) and without (seven studies with thirteen estimates) (Devilbiss et al., 2017; Levine et al., 2018; Li et al., 2018; Nilsen et al., 2013; Surén et al., 2013; Tan et al., 2020; Virk et al., 2016) folic acid food fortification (OR 0.57, 95% CI 0.48–0.68).

### Meta-regression Analyses

Meta-regression analyses were conducted to explore sources for the heterogeneity. China and Europe presented inconsistent results trends (*P* < 0.05) compared with Israel and the USA. The time period of folic acid intake was further explored and the analysis of FA supplements before pregnancy to early pregnancy showed inconsistent trends compared to the results obtained for FA supplements during other supplementary timings (P < 0.05). The analyses suggested that study location and folic acid supplemental timing were identified as sources of heterogeneity. The result of meta-regression analyses was showed in Table 3.

**Table 3:** Meta-regression analysis

	OR	95% CI	*P*
Study design			
Cohort study	Ref	–	–
Case–control	1.69	0.96–2.97	0.060
Study location			
Israel	Ref	–	–
USA	2.96	0.80–11.04	0.098
China	2.50	1.26–4.99	0.013
Europe	7.74	3.20–18.71	< 0.001
Timing of folic acid intake			
During pregnancy	Ref	–	–
Before pregnancy	1.31	0.95–1.81	0.090
Before pregnancy and early pregnancy	0.38	0.18–0.80	0.020
Early pregnancy	0.49	0.17–1.43	0.170
Mode of folic acid intake			
Vitamins and other supplements	Ref	–	–
Folic acid	1.34	0.92–1.96	0.120

*CI* confidence interval, *ref* reference

^a^Amount of heterogeneity accounted for (R2): 93.28%. Residual heterogeneity (I2): 9.68%

### Publication Bias and Heterogeneity

**Fig. 4:**
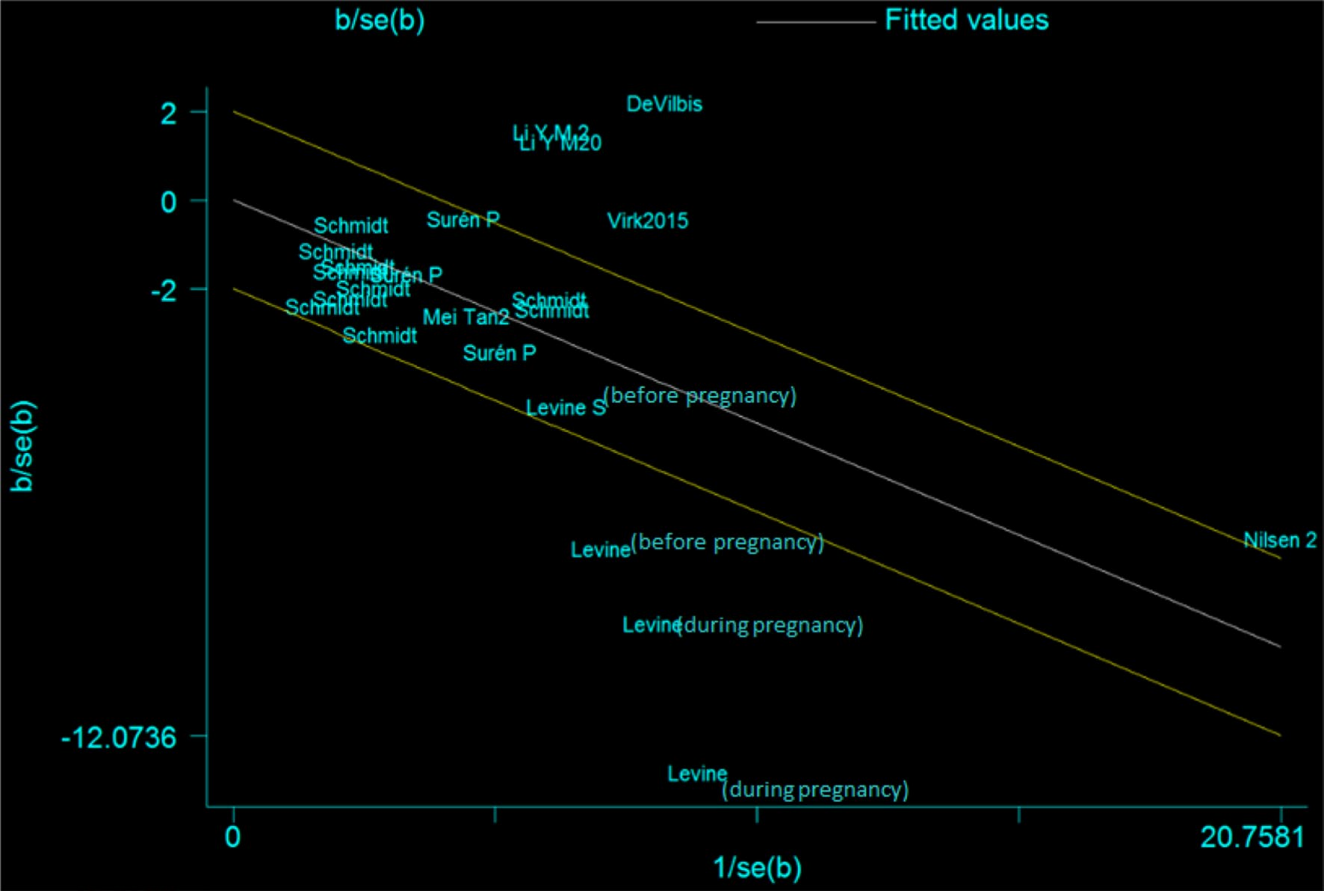
Galbraith plot for heterogeneity

The funnel plot's visual inspection indicated no publication bias (Egger test, P = 0.54, 95% CI 3.27–1.77) (Fig 1 and Fig 2 in the Supplementary Material). In the pooled result for all including studies, evidence of heterogeneity was indicated. Sources of heterogeneity were identified following separate subgroup analysis of the time period, the dose and the modes of folic acid intake (see Fig. 3). Galbraith analyses indicated several studies (Devilbiss et al., 2017; Levine et al., 2018; Li et al., 2018; Virk et al., 2016) had an excessive influence on the pooled result. The result of the Galbraith plot for heterogeneity was shown in Fig. 4.

### Sensitivity Analysis and Influence Analysis

**Fig. 5:**
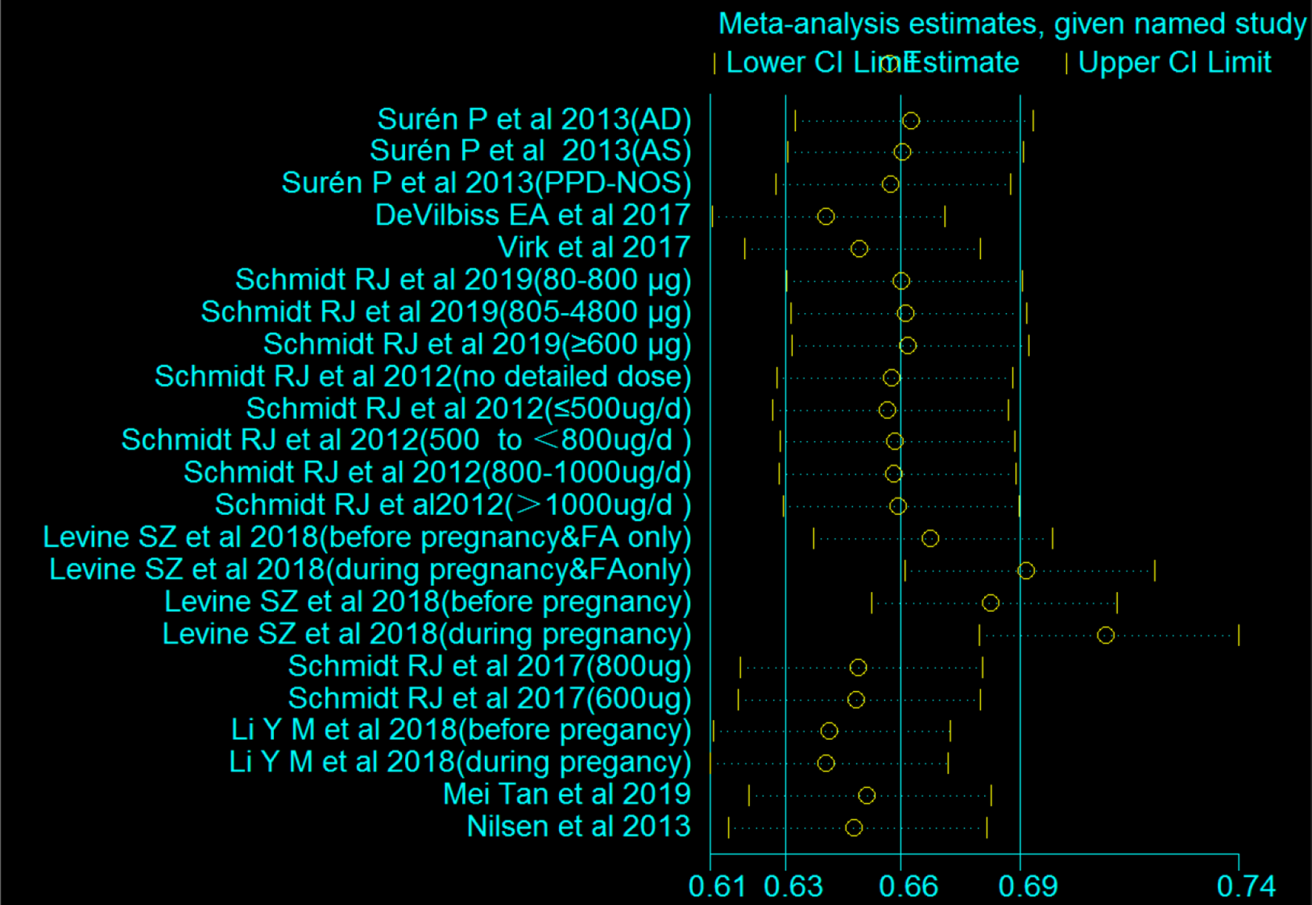
Influence analysis

**Fig. 6:**
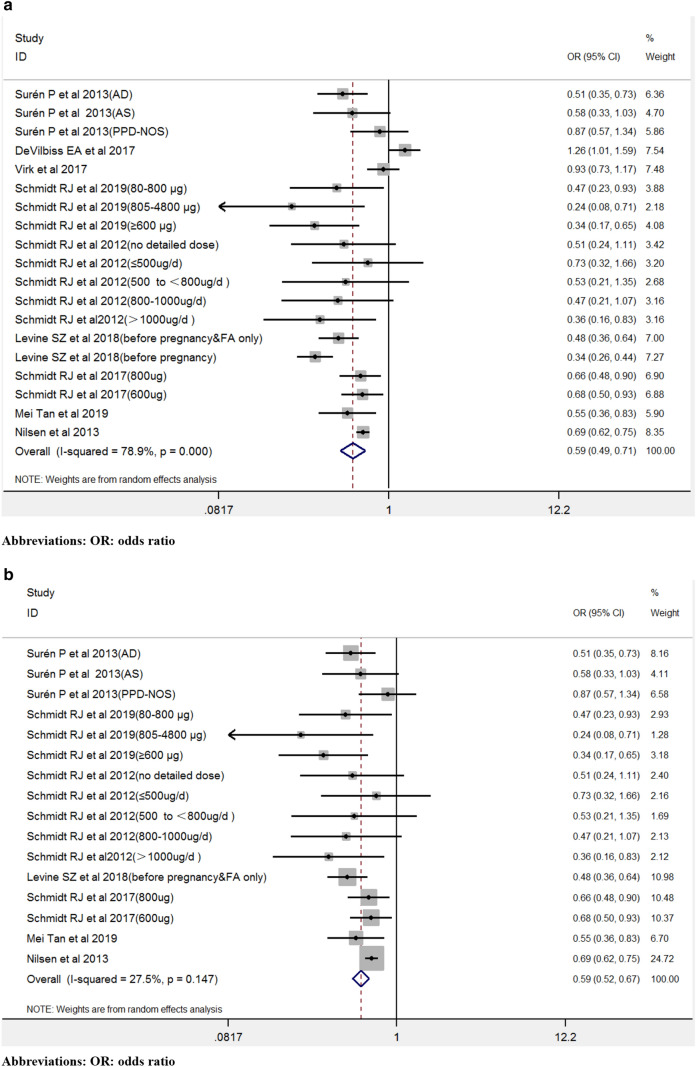
**a** The sensitivity analysis after excluding parital heterogeneous studies. **b** The sensitivity analysis after excluding all heterogeneous studies

Influence analyses indicated that the results were consistent and did not change following iteratively removal of one study (Fig. 5). Figure 6 indicated the sensitivity analysis following the exclusion of several heterogeneous studies. In the present study, heterogeneous studies from Galbraith analyses (Fig. 4) (Devilbiss et al., 2017; Levine et al., 2018; Virk et al., 2016) were not all removed considering the fact that these studies were evaluated as high quality (Table 2). The two sub-studies (during pregnancy) from Israel (Levine et al., 2018) were removed due to highly heterogeneous (Fig. 4) and influence analysis results (Fig. 5). The Li et al., study was also removed due to low quality according to NOS (Table 2) and highly heterogeneous (Fig. 4). Following removal of the three studies, the results didn’t change the pooled results appreciably [OR 0.59, 95% CI 0.49–0.71] but reduced the heterogeneity [I^2^ = 78.9%, P heterogeneity < 0.001] (Fig. 6a). Subsequently, all heterogeneous studies were removed (Fig. 4) (Devilbiss et al., 2017; Levine et al., 2018; Li et al., 2018; Virk et al., 2016), whereas the sensitivity analysis results were retained [OR 0.59, 95% CI 0.52–0.67, I^2^ = 27.5%] (Fig. 6b).

### Quality Effects Model Analysis

**Fig. 7:**
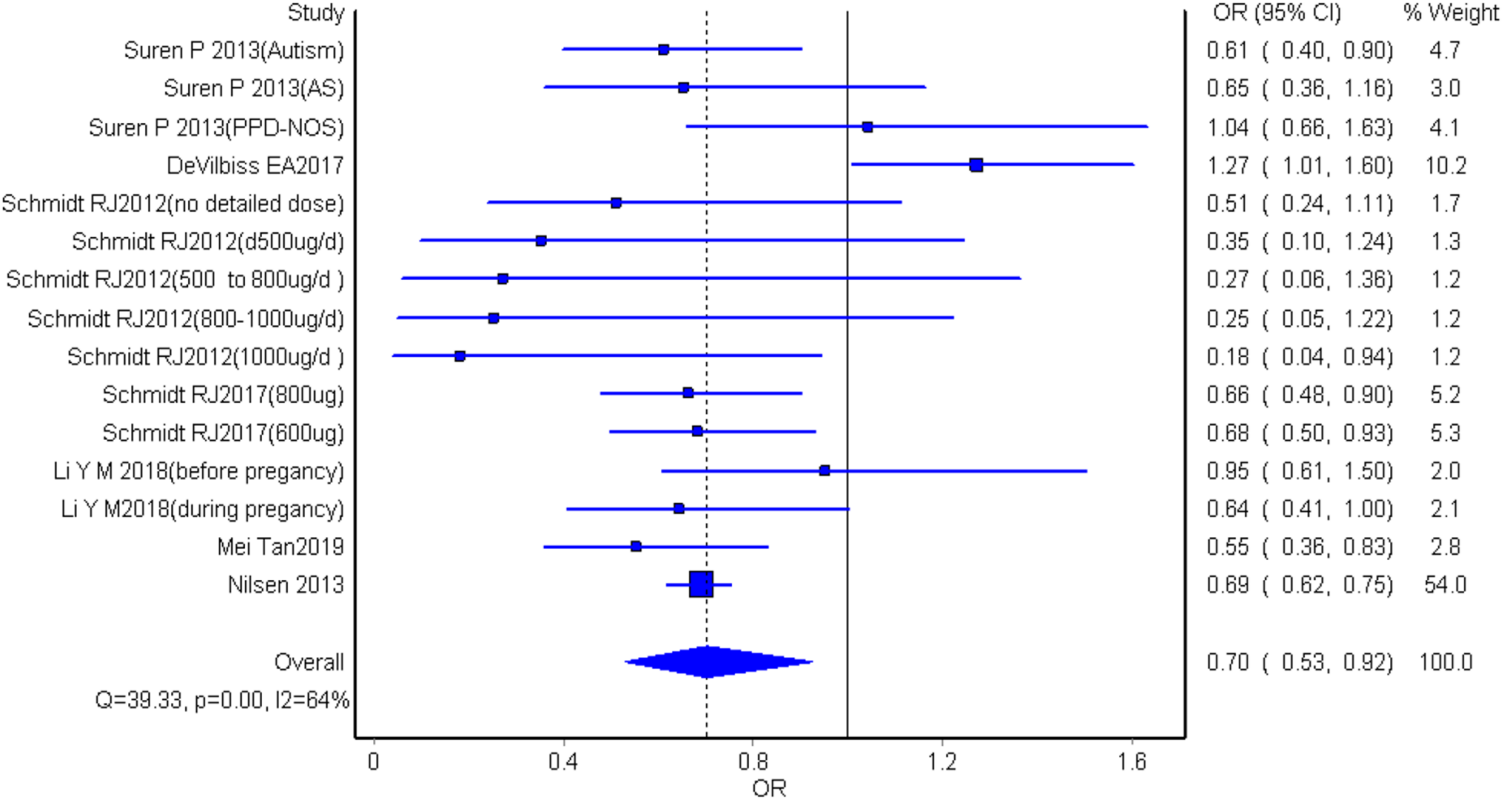
The pooled estimate of ORs in the meta-analyses using updated quality effects

**Fig. 8:**
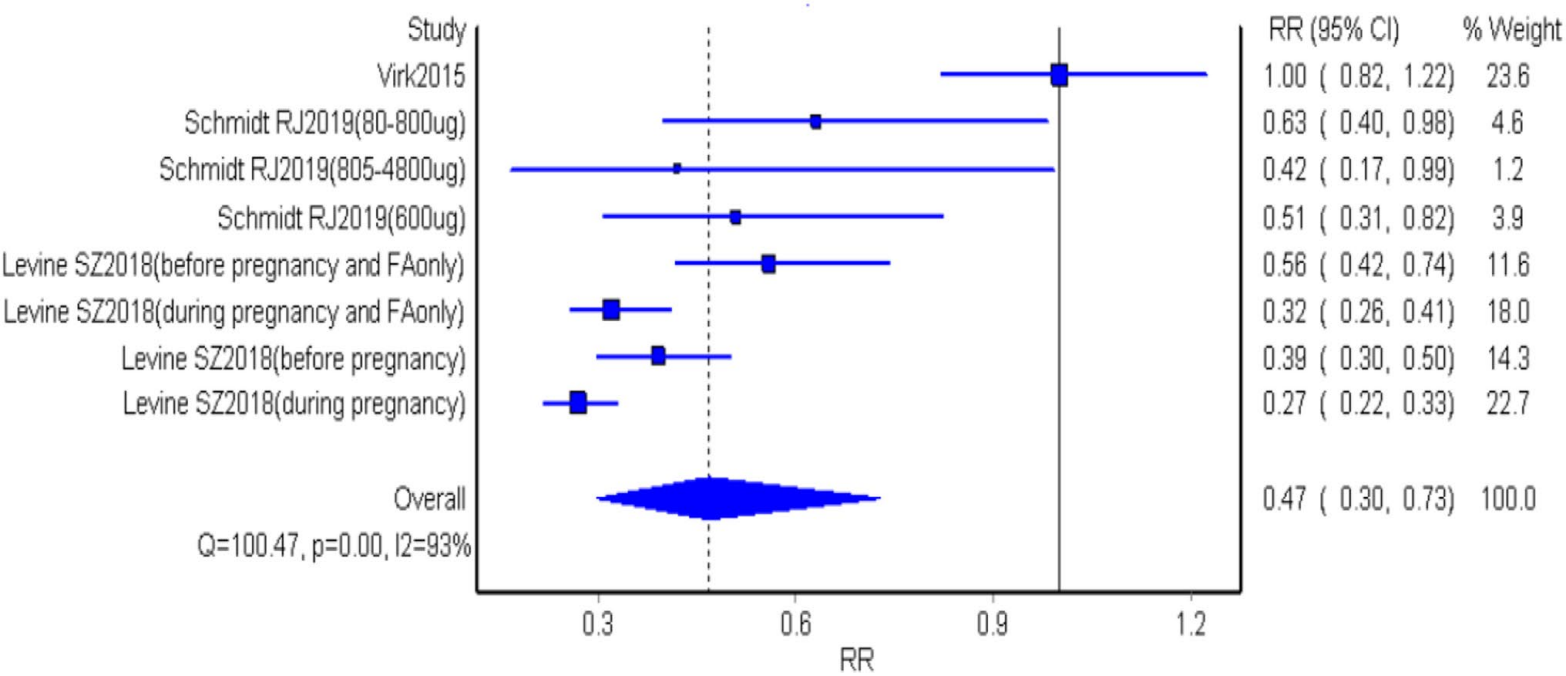
The pooled estimate of RRs in the meta-analyses using updated quality effects

The pooled estimate of ORs (Devilbiss et al., 2017; Li et al., 2018; Nilsen et al., 2013; Schmidt et al., 2012, 2017; Surén et al., 2013; Tan et al., 2020) was 0.70 (95% CI 0.53–0.92), while the pooled estimate of RRs (Levine et al., 2018; Schmidt et al., 2019; Virk et al., 2016) was 0.51 (95% CI 0.31–0.84) in the meta-analysis using updated quality effects. (Figs. 7, 8).

## Discussion

The present study demonstrated that maternal folic acid supplements administered during the prenatal period were associated with 43% lower odds of offspring ASD compared to the subjects without maternal folic acid supplement exposure. Maternal folic acid supplements in early pregnancy may be the sensitive window to reduce the risk of offspring’s ASD. The minimum dosage of folic acid estimated to at least 400 μg daily may provide a protective effect on reducing the risk of offspring ASD. Folic acid intake and other nutrients or folic acid intake can reduce the risk of ASD in the offspring. The preventive effect of maternal folic acid supplements on offspring’s ASD during the prenatal period was explored in countries with or without folic acid food fortification. These findings provided evidence to support the critical role of maternal folic acid supplements in early pregnancy in reducing the risk of offspring ASD. The understanding of the sensitive supplement timing window and the suitable dosage of folic acid exposure may provide evidence for precise intervention and prevention.

Folic acid supplements consumed in early pregnancy or before pregnancy to early pregnancy could reduce the risk of offspring’s ASD (Fig. 3). These findings aid the identification of the optimal efficacy of folic acid in the protection against ASD development. Early pregnancy, notably in the first two months of pregnancy, is a critical period for the central nervous system development, mainly consisting of proliferation and migration of neural progenitor cells. Folate deficiency during early pregnancy may disrupt the proliferation and migration of neural progenitor cells by impairing the efficiency of DNA methylation (Crider et al., 2012; Roffman, 2018) and resulting in brain abnormalities that may be associated with ASD (Choi, 2018; Cusick & Georgieff, 2012; Lieberman et al., 2019; Roffman, 2018). Previous studies indicated that the period from deficiency to the recovery of serum folate levels should be estimated from several weeks to months. Early pregnancy folic acid supplements and periconceptional folic acid supplements should be emphasized and recommended to women of reproductive age. This hypothesis is supported by the sub-analyses (Fig. 3) and meta-regression analyses (Table 3). The preventive effect of maternal folic acid supplements on offspring ASD before pregnancy (Levine et al., 2018; Li et al., 2018) or during pregnancy (no detailed pregnancy period) (Levine et al., 2018; Li et al., 2018) against offspring’s ASD was not observed in the sub-analyses due to a lack of power due to lack of statistical power, which was attributed to the small number of studies. It is important to note that folic acid supplementation before pregnancy reduced the risk of neural tube defects (1991; McGarel et al., 2015; Valera-Gran et al., 2014). Periconceptional folic acid supplements are widely considered to provide sufficient reserves for reducing the risk of neural tube defects and other neuropsychiatric risks (Roffman, 2018). The meta-regression analyses used in the present study verified further the protective effects of folic acid supplementation before pregnancy to early pregnancy on the offspring’s ASD. Although insufficient evidence was reported to support the hypothesis that maternal folic acid supplementation before pregnancy reduces the risk of offspring's ASD, the result should be considered with great caution due to the small number of studies. Future population-based studies are urgently required to explore the most sensitive time-window periods of maternal folic acid intake for the preventive effect on the offspring ASD.

Optimal folic acid dosage is essential for the pregnant woman health, safe preconception and for the development of the fetus. The meta-analysis focused on the exact dosage of maternal folic acid intake and indicated very low heterogeneity (Fig. 3). In addition, our subgroup analysis indicated that the risk of offspring’s ASD reduced from the onset when more than 400 μg folic acid was ingested daily. Maternal folic acid intake above the amount (≥ 400 μg) was already associated with lower ASD risk, as determined from the subgroup analysis. Recent studies reported that higher unmetabolized folic acid (UMFA) in cord blood at birth or maternal blood during pregnancy was associated with an increased risk of ASD (Egorova et al., 2020; Raghavan et al., 2018, 2020). The Boston Birth Cohort Study further observed that low and high folate concentrations in maternal plasma were associated with a higher risk of ASD, presenting a U-shaped risk curve (Raghavan et al., 2018). During the prenatal period, high maternal folic acid exposure demonstrated several behavioral changes in an animal model, such as embryonic growth delay, memory impairment, anxiety-like behaviors, and methyl metabolism changes, which impacted ASD development (Bahous et al., 2017). In addition, harmful effects on brain development were evident, which were due to higher folic acid intake. Therefore, it is necessary to explore the appropriate dosage of folic acid supplementation. Folic acid supplements are absorbed and can be rapidly converted to the 5,10-methylenetetrahydrofolate bioactive form (5,10-methlyTHF) by dihydrofolate reductase (DHFR) and methylenetetrahydrofolate reductase (MTHFR). DHFR in humans has sufficient capacity to efficiently metabolize the appropriate dosage of folic acid (upper intake level:1000 μg/day) (Bailey & Ayling, 2009; Obeid et al., 2016). A randomized controlled trial also pointed out that low or undetectable concentrations of UMFA in serum of mothers and newborns were detected when folic acid intake was at a dose of 400 μg/day during pregnancy (Pentieva et al., 2016). We found that maternal folic acid intake above the amount (≥ 400 μg) had already been associated with lower ASD risk from the subgroup analysis. We also observed that the dose above 500 or 800 μg/day did not offer additional benefit in the protection against ASD development compared to that noted at the dose higher than 400 μg/day. The safety of higher doses of maternal folic acid supplements had been reported (Krishnaveni et al., 2014; Patel & Sobczyńska-Malefora, 2017; Raghavan et al., 2018), and unintended negative consequences of excessive supplemental folic acid should be considered. Furthermore, increased maternal blood or fetal cord blood folate concentrations and an increased risk of ASD should be interpreted fully considering the folate metabolism abnormalities. When folate is present in the body, it is transported into the fetus and the brain primarily by the folate receptor alpha (FRα) mechanism (Desai et al., 2017). Autoantibodies have been identified that can block folate binding to FRα (Desai et al., 2017). These autoantibodies are highly prevalent in children with ASD and their mothers (Frye et al., 2017). Abnormal function of the FRα transport mechanism can interfere with the transportation of folate into the brain and the fetus, potentially generating higher blood folate concentration levels, particularly if folic acid is being supplemented (Frye et al., 2017). Folinic acid, a reduced form of folate, can be transported by the reduced folate carrier (RFC) when the FRα transport mechanism is disrupted byautoantibodies (Frye et al., 2013). Folinic acid supplements have been proven to provide adequate folate to the brain and markedly improve the core associated ASD symptoms in children with ASD (Frye & Rossignol, 2014; Frye et al., 2018). Folinic acid supplementation during the preconception and pregnancy period may be able to prevent offspring’s ASD. Future studies should not only focus on identifying the optimal dose of folic acid supplements to reduce the risk of offspring’s ASD but also on the difference in efficacy between folinic acid and folic supplements for the prevention of the development of offspring ASD during pregnancy.

Subgroup analysis indicated that either folic acid intake alone or a folic acid intake with other nutrients could reduce the risk of offspring’s ASD (Fig. 3). Other nutrients included multivitamins, nutrient specific vitamins, or minerals in the meta-analysis study. The present study demonstrated that folic acid intake only or folic acid intake with other nutrients could protect against offspring’s ASD development. The data did not suggest that folic acid intake with other nutrients had the advantage to reduce offspring's ASD than folic acid intake only. Adequate maternal nutritional status is critical for fetal brain development. In the rapid development period, the brain has heightened sensitivity to nutritional deficiency, which may predispose the fetus to postnatal neurodevelopmental disorders (Stephenson et al., 2018). Additional population-based studies should rigorously investigate whether there is an independent or synergetic effect is evident between folic acid and other nutrients, which could drive the association with reduced ASD risk. In addition to folic acid (an oxidized form of folate), reduced folate forms, such as folinic acid, can be investigated with regard to the possible preventive effects on reducing offspring’s ASD. Unlike folic acid, folinic acid can enter the folate cycle without being converted by DHFR and MTHFR. Folinic acid can also pass the blood–brain barrier by RFC when the FRα is bound to FRAAs or is dysfunctional (Desai et al., 2016; Frye et al., 2013). In a randomized, double-blind placebo-controlled trial, folinic acid supplements were shown to improve verbal communication in children with ASD (Frye et al., 2018). Whether folinic acid supplements during pregnancy prevent the offspring’s ASD has not been previously investigated. Future studies should explore whether folinic acid supplements may have an advantage over folic acid.

The present study explored whether countries and supplementary timings may be significant heterogeneity sources from meta-regression analyses (Table 3). Regardless of the folic acid food fortification policy implemented in countries, maternal folic acid supplements are associated with the reduced risk of children's ASD in the present study. The protective effect of maternal folic acid supplements was consistent in the US and Israeli populations, but not in the European or Chinese people. Several factors can be considered to account for the discrepancy in the results. Initially, the majority of European or Chinese studies did not report the consuming dose of FA intake, which may never reach the specific threshold of folic acid supplements to reduce the offspring’s ASD risk. Secondly, folic acid supplementary timing was a source of heterogeneity that suggested a sensitive period for maternal folic acid supplementation on the prevention of the offspring ASD.

To the best of our knowledge, this is the most comprehensive systematic review and meta-analysis regarding prenatal folic acid supplements and offspring’s autism spectrum disorders. In addition, it is the first to systematically account for supplementary timing, dose, supplemental folic acid mode, and folic acid food fortification. A series of sensitivity analyses were conducted in order to verify the stability and robustness of our results.

Several limitations should be mentioned with regard to the current study. Firstly, a recall bias was present since detailed information regarding maternal supplement intake before and during pregnancy was acquired using questionnaires. However, the majority of studies included in the meta-analysis focused on the analysis of continuous exposure over time rather than a single point time, which reflected a conservative estimate for ongoing folic acid supplement intake status (Schmidt et al., 2012, 2017, 2019; Surén et al., 2013; Virk et al., 2016). Secondly, given the observational nature of the included studies, residual or unmeasured confounding factors are possible. Finally, a significant heterogeneity was observed in this meta-analysis. However, the heterogeneity was settled by subgroup analysis, meta-regression and a series of sensitivity analyses. The location of the study and the timing of folic acid intake were the significant sources of heterogeneity. The aforementioned variable factors should be considered to design future research studies exploring the association between maternal folic acid supplementation and ASD.

## Conclusions

Our systematic review and meta-analysis provided the new insight that maternal folic acid supplementation during the prenatal period, notably in early pregnancy in the reduction of the risk of offspring ASD. The consumption of a minimum daily amount of 400 μg folic acid from dietary supplements may be associated with the reduced risk of offspring ASD.

## Supplementary Information

Below is the link to the electronic supplementary material.

Supplementary file1 (DOCX 167 kb)
