# Supplementary material for: Prenatal Folic Acid Supplements and Offspring’s Autism Spectrum Disorder: A Meta-analysis and Meta-regression
Source: J Autism Dev Disord. 2021 Mar 20;52(2):522–39. doi: 10.1007/s10803-021-04951-8 (PMC8813730; doi:10.1007/s10803-021-04951-8)
Supplement: Supplementary file 1 — Supplementary file1 (DOCX 167 kb) [file 10803_2021_4951_MOESM1_ESM.docx]

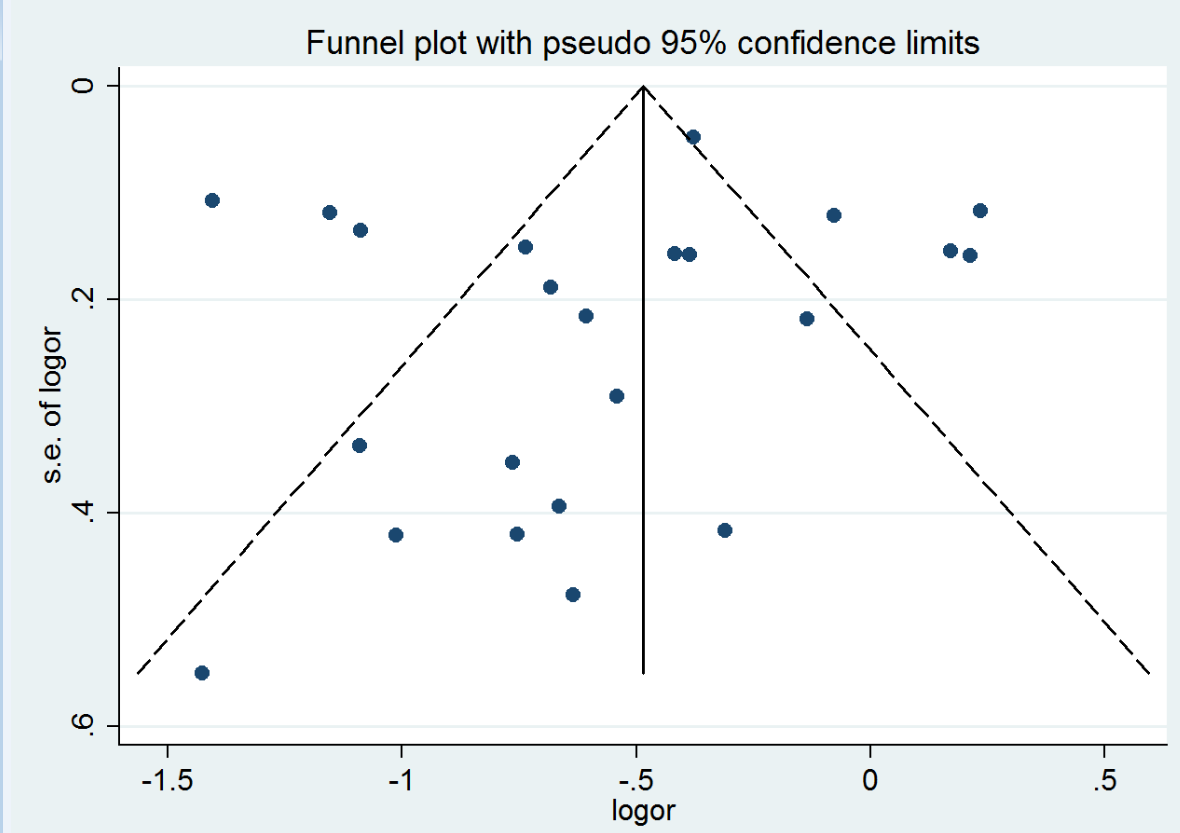


**Fig.1 The funnel plot of all included studies showing the number of potential missing studies**


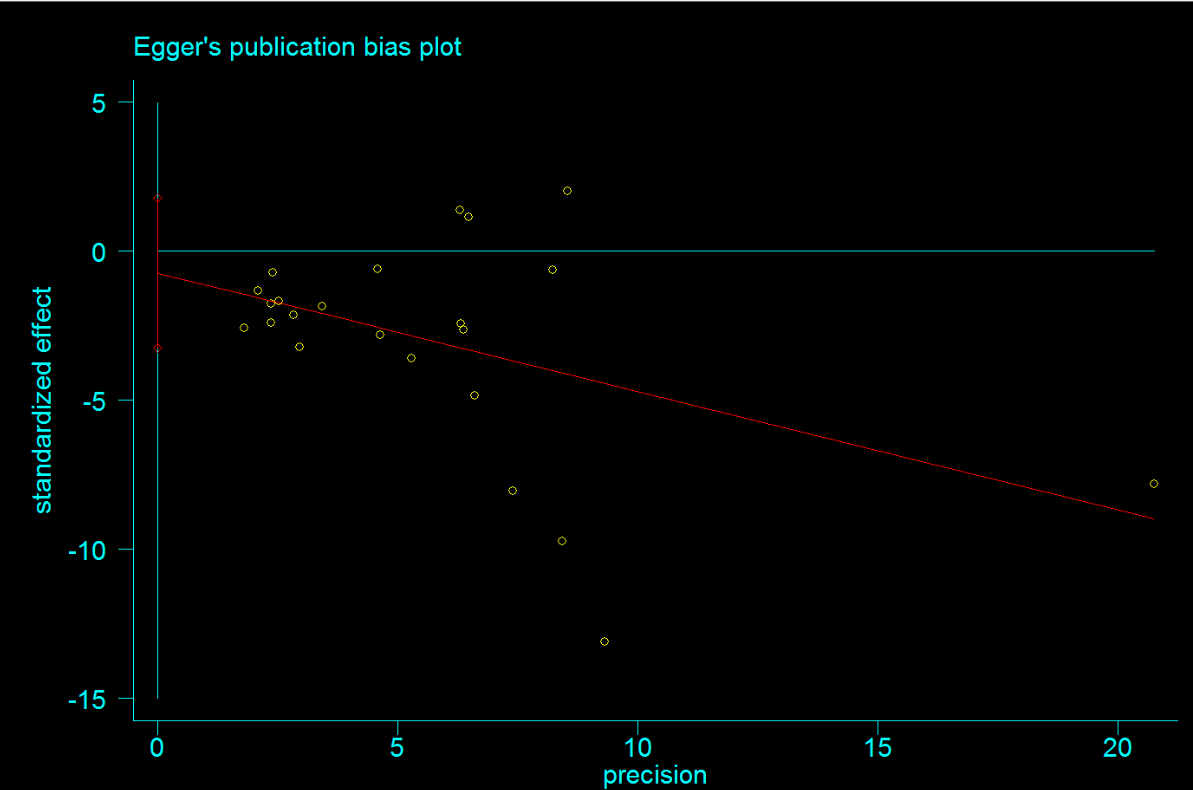

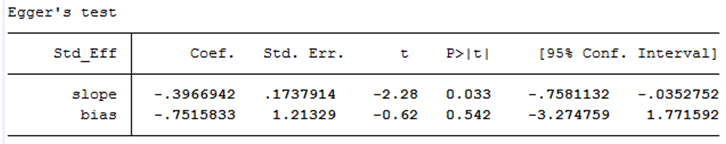


**Fig.2 Egger's publication bias plot**
